# Co-Designing the PATH Harm Reduction Training for the Peer Workforce: A Community-Based Participatory Approach

**DOI:** 10.21203/rs.3.rs-8138704/v1

**Published:** 2025-12-11

**Authors:** Garland Gerber, Dennis P. Watson, Keoni K. Bermoy, Lisa D. Taylor, Ashli J. Sheidow, Patrick F. Hibbard, Justin S. Bell, Adam Caba, Heather Ogdon, Samuel Peterson, Robert J. Richard-Snipes, Venus Staton

**Affiliations:** Northeastern University; Chestnut Health Systems; Chestnut Health Systems; Chestnut Health Systems; Chestnut Health Systems; Chestnut Health Systems; Chestnut Health Systems; AND Services, LLC; Heartistic Recovery, LLC, Hadden Township; Four Rivers Behavioral Health; Greater Nashua Mental Health; Alaskan AIDS Assistance Association

**Keywords:** Harm Reduction, Peer Supports, Recovery Rervices, Community-Based Participatory Research, Community Board, Co-design, Behavioral Health Workforce

## Abstract

**Background:**

Opioid use disorder and related overdoses remain a leading US public health challenge. Peer Recovery Specialists (PRS), individuals with lived experience who support others in recovery, frequently deliver harm reduction services but face high job demands, stigma, and role conflict due to abstinence-oriented norms. These stressors contribute to burnout, turnover, and diminished service quality. Despite growing recognition of PRSs’ role in harm reduction, few interventions address their workforce development or resilience needs. To strengthen harm reduction delivery and workforce sustainability, we developed Peer Advanced Training in Harm reduction (PATH), a blended educational intervention co-designed with community partners using a Community-Based Participatory Research (CBPR) framework.

**Methods:**

PATH was designed to enhance PRSs’ personal and job-related resources through (1) three one-hour eLearning modules that apply adult learning and narrative storytelling to shift stigmatizing attitudes and strengthen harm reduction skills, and (2) an ECHO–based case discussion series to build confidence, policy literacy, and organizational alignment. A Community Board (CB) of eight members with lived recovery, harm reduction, and related service experience guided iterative content prioritization across six meetings (February–June 2025). CB members brainstormed, ranked, and refined eLearning and ECHO topics through surveys and facilitated discussions. Cultural exchange surveys assessed perceptions of collaboration, communication, and trust between CB members and researchers at two time points.

**Results:**

CB deliberations identified relational harm reduction—emphasizing empathy, authenticity, and participant autonomy—as the unifying theme across all training modules. Four final eLearning modules emerged: Embracing the Harm Reduction Approach, Integrating Harm Reduction into Participant Recovery Plans, Supporting PRSs to Do Harm Reduction Work, and Advancing Harm Reduction. ECHO priorities were also refined to reflect the pragmatic nature of peer work as perceived by CB members. Cultural exchange ratings were uniformly high (mean > 5.5 on a 6-point scale), reflecting strong mutual respect, shared learning, and perceived impact.

**Conclusion:**

Through CBPR co-design, PATH demonstrates how centering people with lived/living experience can yield a culturally grounded, practice-ready workforce intervention that strengthens harm-reduction capacity and sustainability. Findings establish feasibility and guide the forthcoming pilot and subsequent clinical trial to assess PATH’s impact on PRS workforce outcomes and service quality.

## BACKGROUND

Substance use disorders (SUD) remain a major US public health threat, contributing to an estimated 220,511 deaths from October 2023 to September 2024, with 40% being attributed to opioids [1]. For people who use drugs (PWUD), harm reduction is the first line of overdose defense. Expanding access to harm reduction services requires supporting the often underprepared and under-supported workforce that delivers them [2–5]. These individuals work in stressful conditions associated with a high burnout risk [6, 7], which can lead to poorer job performance and lower service quality [8, 9]. Peer Recovery Specialists (PRS)—professionals with lived recovery experience who provide mentoring, coaching, and social support—may be a particularly vulnerable class of harm reduction workers.

PRSs frequently deliver or intersect with harm reduction services [10–12]. Yet, the demanding nature of their work raises workforce sustainability concerns. PRSs often experience elevated job stress, which is concerning given their status as people who are also managing their personal SUD recovery [13]. A recent scoping review found wide-ranging negative workforce outcomes associated with PRS work in the literature—including burnout, vicarious trauma, compassion fatigue, turnover intention, and return to uncontrolled substance use [14, 15]. These risks may be heightened in harm reduction service settings, where workers frequently encounter situations exposing them to vicarious trauma, stress, and stigma toward people who are or have ever used substances [7, 16, 17]. Systemic (e.g., drug criminalization, abstinence-oriented policy) and organizational (e.g., marginalized roles, limited training) barriers can further contribute to stress by impeding harm reduction services delivery [2, 3]. Because many states require abstinence for PRS certification, peers may also enter practice with abstinence-aligned orientations that can result in conflict with harm reduction-focused roles. For instance, one survey (N = 260) indicated that endorsing abstinence-only treatment was associated with 55% lower odds of supporting harm reduction-focused safe consumption sites [18]. Even when PRSs and other clinicians endorse harm reduction, uptake remains challenging due to limited guidance and individualized outcome expectations [19, 20], and harm reduction policies alone do not ensure service integrity [19, 20]. These misalignments may foster “don’t-ask–don’t-tell” environments that obscure recovery pathways and increase substance-use–related risk [19, 21].

Strengthening PRSs’ personal and job-related resources can help prevent burnout and improve harm reduction service delivery [22]. Evidence from PRS and harm reduction studies shows that increased resources (e.g., coping strategies, clear roles, and supportive supervision) can mitigate burnout, enhance job satisfaction, and promote retention [23, 24]. Appropriate supports may also help PRSs reframe job demands as meaningful challenges, enabling “job crafting” to optimize work conditions and identify small but important client improvements [19, 20, 22, 25]. Because continuing education is essential for developing these competencies [5, 26], targeted approaches are needed to build harm reduction delivery skills, counter abstinence-informed beliefs that might undermine harm reduction efforts, and ensure alignment between policy and supervision [19, 20, 25, 27, 28]. Educational strategies have demonstrated effectiveness in improving key personal and organizational resources that could reduce burnout and turnover among PRS and harm reduction workers [29–33].

To address these challenges, we designed Peer Advanced Training in Harm reduction (PATH) as a workforce intervention to enhance the personal and job-related resources PRSs need to manage job demands, reduce burnout, and strengthen harm reduction service delivery. Rooted in the Lead Investigator’s (DPW) prior work [5, 19, 34], PATH integrates two core strategies: (1) virtual education, delivered through one-hour modular eLearning content incorporating adult-learning methods and narrative case stories to build harm reduction knowledge, shift stigmatizing attitudes, and support organizational harm reduction integration; and (2) an ECHO model [35], a form of case-based group training demonstrated to improve clinical and community impact [36] to develop PRSs’ skills further and assist supervisors and administrators in strengthening harm reduction-aligned policies and supports. Collectively, PATH was structured as a 15-hour blended program (3 hours of eLearning and 12 hours of ECHO participation over approximately 3 months).

This manuscript describes the Community-Based Participatory Research (CBPR) approach we used to refine PATH’s eLearning and ECHO content (information about the larger PATH study can be found at https://pathstudy.info/). The strategy centers on researchers’ partnership with a Community Board (CB) —a group comprised of people with lived/living experience (PWLLE) in recovery, PRS service delivery, and harm reduction—who provided structured guidance throughout intervention development. We followed this approach because it promotes shared decision-making and trust between researchers and affected communities [37–39], helping ensure our work centers the lived expertise of people in recovery and PRS throughout the research process [39–42]. To address common risks in CBPR work, such as power imbalances, failure to recognize lived expertise, and tokenism, the researchers integrated iterative feedback structures and engaged CB Members early to ensure authentic rather than symbolic participation [43, 44, 44–47]. Through this collaboration, PATH’s CB is shaping content and the broader study to reflect the realities of PRS practice and lived expertise, ensuring authenticity in the narratives, examples, and strategies being developed. As demonstrated below, working with the CB helped ensure PATH content reflected community priorities and supported PRS capacity to deliver harm-reduction services. In the spirit of this work, researchers and CB Members co-authored this manuscript.

## METHODS

### Community Board Member Recruitment Process

Following methods detailed in an online toolkit [48], the Research Team recruited CB applicants via an email and flyer, with targeted distribution to 19 US-based harm reduction and peer organizations. Interested individuals were asked to complete a brief online application, which collected demographics, including location of primary residence; personal or professional experience with harm reduction, criminal-legal system, and mutual aid groups; professional experience with recovery organizations; personal experience with recovery; and details of how experiences would inform work as a Board Member. Following a three-week recruitment period, the team comprising six researchers (two with lived experience) and three Community Partners (all with lived experience) screened applications for initial eligibility, scoring them using a 3-point scale applied to three specific factors: harm reduction experience (1/”no experience”, 2/”delivered or received harm reduction services”, 3/”delivered and received harm reduction services”), recovery or harm reduction pathway (1/”not in recovery or not actively using harm reduction”, 2/”in recovery or actively using harm reduction”, 3/”considers self in recovery and is actively using harm reduction”), and recovery work experience (1/”no experience working in a recovery setting”, 2/”experience working in at least one recovery setting”, 3/”experience working in more than one recovery setting”). Applications were then sorted by average score, region of residence, and overall completeness. Two members of our research team then reviewed the highest-scoring applicants from each region and selected individuals for follow-up calls based on overall appropriateness, experience, and completeness of their applications. Each call was conducted by two research team members using a structured script. Researchers also took notes pertaining to each section of the script. After the call, the interviewers created a consensus score for the applicant using a four-point scale: 1/”very poor fit”, 2/”poor fit”, 3/”good fit”, 4/”strong fit”. In the final stage, the entire team of researchers met to review the candidates, weighing final selection based on scores as well as demographic and geographic diversity.

### Community Board Meetings

Board meetings were held virtually via Zoom. They were co-facilitated by the study’s Principal Investigator and a Board Chair who was elected by the other members (a co-Chair who helps facilitate meetings in the Chair’s absence was also elected). Meetings are not recorded to ensure CB Members are comfortable sharing their lived experience; however, the study’s Project Manager takes detailed notes that are distributed by email after each meeting. CB Members are also asked to complete tasks between meetings, which researchers aim to limit to no more than 30 minutes. Per the recommended ethical guidelines for facilitating CBs [49], Members are compensated for time spent in meetings and completing tasks, and submit monthly invoices to the research team.

The content prioritization process spanned 6 meetings from 2/19/2025 to 6/2/2025. Our approach was adapted from a three-step process for prioritizing research priorities with CB Members previously developed by the JEAP Initiative as part of its goals to advance addiction recovery science [48]. We applied this three-step process separately for (a) eLearning modules, (b) eLearning lessons, and (c) ECHO training topics. **Brainstorming** (Step 1) was accomplished over one meeting through a combination of guided discussions and breakouts. The meeting began with researchers providing an overview of the topics proposed in the original grant application. Researchers then organized the Board into breakout rooms of 2–3 members and asked them to review the content and brainstorm suggestions, including what topics to drop or add and what content should be covered as part of the topics. The Board then reconvened, and each breakout group provided a report back to the larger group. **Prioritization** (Step 2) was completed using an online survey delivered between meetings. CB Members were asked to rank the importance of each topic, provide additional topic suggestions, and use text boxes to specify any content they believed was necessary to cover. **Refinement** (Step 3) occurred in a meeting following the prioritization survey. Researchers presented a summary of the survey results, and CB Members provided their final feedback through open discussions. The three-step process was completed separately for eLearning modules (2/19/25 to 3/3/25), eLearning lessons (3/3/25 to 3/19/25), and ECHO training topics (5/7/25 to 6/2/25), and the eLearning module results informed the initial brainstorming for the eLearning lessons. These activities are not considered human subjects research, as CB Members contributed their expertise to intervention development as paid consultants.

### Cultural Exchange Survey

We used an adapted version of the cultural exchange survey to assess CB Members’ perceptions of collaboration and communication related to the prioritization process [50]. The survey was developed to assess knowledge, attitudes, and practices exchanged between individuals from different organizations collaborating to implement an evidence-based practice [51]. The instrument was previously adapted by the JEAP Initiative to assess research relationships from the perspective of Community CB Members. The adapted instrument includes 22 questions that CB Members are asked to rate on a scale from 1/”not at all” to 6/”a great deal”: four questions focus on the CB Members’ understanding and commitment; three questions focus on their relationships with other CB Members; and 13 questions focus on their relationships with researchers. There are also two open-ended questions where respondents can comment on collaborations with fellow CB Members and researchers, respectively. We also included two additional questions developed by the CB Chair to assess respondents’ perceptions of the CB recruitment process and desire to continue participation in CB activities. All surveys were anonymous. All questions are listed in Table 4.

We administered the survey electronically at two time points: immediately after the meeting’s finalization (1) the eLearning lesson content and (2) the ECHO training topics. Cultural exchange surveys are conducted anonymously with the Board, with no ability to connect completed surveys to respondents. As such, we obtained a waiver of consent for this activity (Chestnut IRB #2025–52), and consent was not required for data collection.

Our analysis summarized the survey data using descriptive statistics (mean and standard deviation), and we calculated differences in average scores across surveys to assess whether perceptions of the CB’s collaboration or communication changed. We also summarized open-ended responses.

## RESULTS

### Final Board Member Selection

Table 1 presents the characteristics of the CB Applicants, those who completed follow-up calls, and the final selected CB Members. Eighty-nine completed the recruitment survey within the initial three-week application window. Researchers invited 23 total applicants to participate in follow-up calls. Of these, five did not reply to our requests, four no-showed the scheduled call, and 14 completed the call. Of the original eight candidates selected, one declined participation, and an alternate was chosen in their place. Each of the eight selected individuals participated in an initial onboarding meeting, during which we described CB expectations, meeting schedules, and compensation, and then completed expert consultant paperwork that the researchers had modified from standard organizational templates specifically for CB members.

### PATH Content Prioritization Results

#### eLearning prioritization results.

The initial module brainstorming discussion included six CB Members. The discussion focused on the need for clarification regarding the modules proposed in the original grant application, including what the content of each module might look like. After the Principal Investigator answered their questions, CB Members voiced that all three original topics included in the grant application were important. After the breakout session following this discussion, one group reported that they discussed the importance of harm reduction advocacy. They defined this as including information to support advocacy efforts of (a) recovery organizations and (b) individual peers and (c) teaching service participants to advocate for their individually chosen recovery path, particularly if it includes harm reduction.

The (a) module and (b) lesson prioritization surveys were delivered separately. Both surveys included the three original module topics and lessons and the newly suggested topic of harm reduction advocacy. Results are shown in Table 2. All 8 CB Members completed the module prioritization survey, and 7 completed the lesson prioritization survey. The average scores indicated that CB Members found all four suggested Module topics to be of high priority. History of Harm Reduction and the Compatibility of Harm Reduction with the 12 Steps were the only lesson topics that received an average rating below 3, indicating they were of less priority for CB members.

All 8 CB Members attended the (a) module refinement discussion, and 6 attended the (b) lesson refinement discussion. At each meeting, the Principal Investigator presented the prioritization results and a summary of open-ended feedback from the relevant prioritization survey conducted prior to the meeting. Afterwards, the CB Members verbally affirmed their agreement with the scores and the qualitative summaries, as well as expanded on information that they thought needed to be covered. Discussions around the newly proposed advocacy module and its corresponding lessons were less concrete, suggesting this module might require more focused development than the others. These discussions also led to the rewording of five lesson titles to reflect their adjusted focus, as shown in the far-right column of Table 2. The rest of this section presents a combined summary of the discussion results from both meetings as they pertain to each module and its corresponding lessons.

CB Members reached a clear consensus on the importance of beginning the training with a strong foundation in harm reduction philosophy and practice. They agreed that Module 1, *Overview of the Harm Reduction Approach*, should orient learners to the central tenets of harm reduction, address common misconceptions, and contextualize the approach relative to abstinence-based and recovery-oriented models. They emphasized that Lesson 1a, *History of Harm Reduction*, should emphasize the historical roots of harm reduction, including its emergence in response to the HIV/AIDS epidemic and its application across diverse populations. Lesson 1b, *Harm Reduction Principles*, was discussed as needing to focus on the foundational philosophical and ethical principles of harm reduction, such as autonomy, non-coercion, and compassion, and that the content should be guided by the National Harm Reduction Coalition’s 8 Guiding Principles [52]. CB Members decided that Lesson 1c should be changed to be *Introduction to Relational Harm Reduction*—an approach that centers the quality of the relationship between a service provider and a person who uses drugs as a core mechanism of support and change [53]—and that the original stages of change topic should be combined with the Module 2 lesson on motivational interviewing. This discussion also pointed to the need to focus the lesson mostly on the stages through which behavioral change is theorized to occur and how it can be applied within the context of harm reduction. This was because (a) most peers are already familiar with the stages of behavior change within an abstinence framework, (b) Motivational Interviewing is too large a topic to cover adequately in an eLearning format, and (c) most peers have already received Motivational Interviewing training. CB Members expressed that the concept of relational harm reduction, which none had been introduced to prior to their work on the PATH CB, was so important that it needed to be woven throughout the rest of the training after its introduction. Finally, although it received a lower prioritization score than other lessons, the Board affirmed the value of Lesson 1d, *Compatibility of Harm Reduction with the 12-Steps*, to challenge stigma and broaden perspectives around 12-step compatibility and other mutual aid approaches.

CB Members emphasized that Module 2, *Integrating Harm Reduction into Participant Recovery Plans*, was critical for supporting peers in applying harm reduction strategies in direct service contexts. They stressed that the lessons should help peers navigate personal boundaries while fostering nonjudgmental, client-centered communication. They expressed that Lesson 2a, *An Overview of Harm Reduction Strategies*, should introduce core harm reduction strategies and real-world scenarios to support skill-building. As discussed above, CB Members recommended merging information on the stages of behavior change (initially in Module 1, Lesson 3) and making it a greater focus of the lesson, transforming Lesson 2b into *Stages of Change and Harm Reduction*. Lesson 2c, *Talking Openly About Drug Use to Identify High-Risk Behaviors*, was discussed as needing to teach peers how to talk openly about drugs when working with service participants who were engaged in abstinence-focused programs such as drug courts, with careful attention to legal and ethical disclaimers and clarity around peer roles. For Lesson 2d, *Setting Harm Reduction Goals*, CB Members supported a focus on participant-directed goal setting, incorporating SMART (specific, measurable, achievable, relevant, time-bound) goals where appropriate and helping participants understand all available recovery pathways while acknowledging the influence of peer biases in recovery coaching and planning activities. Lesson 2e, *Celebrating Any Positive Success*, was discussed as requiring challenging abstinence-only definitions of success by celebrating incremental progress and alternative markers of improvement, such as reduced use or restored relationships.

The discussion of Module 3, *Supporting PRS to Do Harm Reduction Work*, focused on organizational and supervisory challenges. CB Members agreed this module should be primarily targeted at supervisors and organizational leaders, with a focus on reducing stigma, strengthening supervisory practices, and fostering workplace environments that support harm reduction delivery. CB Members proposed Lesson 3a, *Harm Reduction-Friendly Policies*, center on policies that reinforce the legitimacy of non-abstinencebased outcomes for participants and staff and tensions between harm reduction-friendly policies and the requirements and scope of peer certification. Lesson 3b, *Employee Supervision*, was discussed as needing to address hierarchical dynamics, prevent burnout, and ensure peers are adequately supported through training and equitable workloads. In discussing Lesson 3c, *Identifying and Addressing Community Barriers to Harm Reduction Work*, CB Members identified barriers at the community level—such as denial or stigma—and recommended preparing peers with relevant data and messaging strategies. CB Members emphasized that Lesson 3d, *Speaking with Community Stakeholders about Harm Reduction*, should focus on using narrative stories and evidence when engaging stakeholders, aiming to increase public understanding and organizational buy-in.

In contrast to the other modules, the conversation around Module 4, *Advancing Harm Reduction*, was more exploratory, with CB Members acknowledging the value of the content but recognizing that further development is needed. They agreed the module should empower peers and participants to engage in harm reduction efforts, especially given the widespread misconceptions and resistance. Discussions indicated that Lesson 4a, *Introduction to being a harm reduction champion*, was reframed to address specific strategies for championing harm reduction within organizations and communities, emphasizing the importance of teaching peers about different methods for community education, as well as various harm reduction activities of focus. Lesson 4b, *Strategies for promoting harm reduction*, should serve more as an introduction to advocacy efforts, focusing on the history of advocacy and emphasizing the importance of human rights and dignity. The lesson should provide peers with an overview of evidencebased, participant-centered advocacy approaches. In discussing Lesson 4c, *Supporting Participants’ Voices*, CB Members confirmed a need to support participants who struggle with low self-efficacy or confidence in advocating for their personal recovery or harm reduction path. Further, discussions with CB Members prompted a change in language choice for these lessons, moving away from the term ‘advocacy’ and utilizing ‘championship’ in its place. This decision centered around ensuring training materials feel accessible and resonate with diverse groups of peers, better reflecting peer-led leadership activities that are approachable and do not require policy-level or system-facing work.

#### ECHO prioritization results.

The ECHO prioritization survey included eight ECHO didactic training topics suggested in the grant application. All 8 CB Members completed the survey. The average scores indicated that they found seven of the topics to be of moderate importance. The lowest-scoring topic, indicating it was only considered of slight importance, was *Supporting Colleagues to Deliver Harm Reduction*.

The ECHO topic refinement discussion included 6 CB Members. The discussion resulted in merging two topics, rewording the others to reflect a modified focus, and adding one new topic. These changes are reflected in the far-right column of Table 3.

During these discussions, Topic 1, *Relational Harm Reduction*, emerged as a top priority, with strong support for emphasizing autonomy, empathy, and trauma-informed, dignity-affirming care. Members highlighted the need to focus on building trust with service participants as central to effective harm reduction work. Topic 2, *Managing Boundaries with Clients*, was reconceptualized to include self-care, recognizing that boundaries are a form of self-care and that peers receive exposure to self-care topics from numerous other sources. Discussions emphasized the protective and sustaining function of boundaries for peer workers, especially in smaller communities and dual-role relationships. Self-care was framed as both a personal and organizational responsibility, modeled by supervisors and supported through mentorship. Topic 3, *Supporting Colleagues to Deliver Harm Reduction*, was reframed to focus on creative problem-solving and mutual support in team settings. Though it received a lower prioritization score, CB Members emphasized its value in promoting a nonjudgmental work culture and navigating team stigma, especially around medications for opioid use disorder. Topic 4, *Harm Reduction with Participants Experiencing Homelessness*, was endorsed as a critical topic, with attention to trust-building, field-based engagement, and the navigation of legal and systemic barriers. Topic 5, *Low-Threshold Treatment and Medication-Assisted Recovery*, was updated to reflect a broader focus on medical interventions across substance use disorders. CB Members stressed the need for timely access, reduced barriers, and the importance of framing treatment within participant-centered, evidence-based, and non-stigmatizing approaches. Topic 6, *Harm Reduction in Rural Low-Resource Areas*, was slightly reworded to broaden its relevance while still addressing challenges specific to rural and other under-resourced communities, including transportation, relationship-building, and the use of national harm reduction resources. Topic 7, *Harm Reduction with Criminal-Legal System-Involved Clients*, remained unchanged. Discussion emphasized state-level policy differences, working within mandated systems, and supporting clients in navigating legal requirements while maintaining harm reduction principles. Finally, the new Topic 8, *Harm Reduction Policy and Legal Structure*, was added to address a noted gap in the structural context impacting harm reduction services. This session will provide an overview of relevant laws, historical and emerging policy trends, and advocacy strategies, while maintaining sensitivity to political dynamics across service settings.

### Cultural Exchange Survey Results

Table 4 presents the means and standard deviations for all the Cultural Exchange Survey questions the researchers requested that the CB Members complete. Not all CB Members completed the survey, which is understandable since this was an optional activity to assess CB engagement quality that was outside of the assigned between-meeting prep work. Ratings for all questions indicated strong agreement, and minimal changes between the two time points indicate general stability in opinions.

Open-ended feedback was similarly positive. Comments about working with the other CB Members indicated they enjoyed interactions with each other and have respect for their fellow CB Members’ different points of view and experience:

- “I am pleasantly surprised by how much I enjoy working with everyone on this board. I look forward to every one of our meetings.”
- “Awesome group of people who bring different ideas and knowledge to the table.”
- “I have so much respect for the diversity and expertise of this group!!!!”.

Comments directed toward researchers indicated CB Members appreciate the respect demonstrate toward them, the perspective they bring, and their efforts to explain technical information in an accessible manner:

- “I appreciate the research team’s respect for the variety of experience and circumstances the CB Members have.”
- “The research team is wonderful, I have learned so much about how a project like this comes together.”
- “The research team has done a great job of explaining and relating the info to the board.”

Specific processes that CB Members commented on as being effective included the recruitment process, attention researchers pay to the value of CB Members’ time, the breakout rooms used in meetings, and the time in meetings dedicated to allowing the CB Members to interact without researcher presence:

- “It shows that a lot of thought and work went into the selection through the advertising, interviewing, and selection process.”
- “The constant mindfulness of my time and experience.”
- “The breakout rooms have been a good opportunity to get to know the other board members as well as work on the project.”
- “I like that the research team has now incorporated time for Board to meet just us without the research team.”

Lastly, one comment expressed that a Board Member was struggling with other members’ behaviors, which they expressed demonstrated a lack of participation. However, there was no indication of what these behaviors were.

## DISCUSSION

In this early phase of our work, we examined the development of the PATH curricula through a community-engaged process that centered PWLLE of substance use, harm reduction, and related service provision. Consistent with participatory curriculum design research, centering PWLLE enhanced cultural relevance and acceptability while strengthening trust and shared ownership between researchers and community partners [1–3]. Our study aligns with a national shift, reflected in NIH investments such as the Justice Community Overdose Innovation Network (JCOIN) and the JEAP Initiative, which institutionalize CBPR models embedding PWLLE as core partners [54, 55].

CB deliberations underscored relational harm reduction as a foundational for the entire curriculum. Relational harm reduction recognizes that reducing substance use-related risk is more than just distributing safer-use and overdose-prevention supplies. It prioritizes trusting, nonjudgmental relationships in which providers partner with people who use drugs to promote safety, autonomy, and wellbeing, recognizing that connection itself is a core intervention [53]. Because relational harm reduction centers connection as a core intervention, CB members emphasized that peer services should prioritize authenticity, nonjudgment, and participant autonomy. This aligns with emerging PRS and harm reduction workforce literature that emphasizes trust-building and boundary management as prerequisites to effective service delivery [7, 13, 15, 16, 23, 24, 56]. At the same time, CB members noted ongoing tension between harm-reduction and abstinence-oriented recovery philosophies, highlighting a need for organizational structures that model non-punitive supervision and recognize multiple recovery pathways. CB Members also recommended integrating historical and philosophical context throughout modules to highlight the compatibility of harm reduction and abstinence-based recovery, reducing stigma and bridging cultural divides. These insights parallel studies documenting moral distress and burnout among behavioral health, legal, and harm reduction professionals whose work requires them to navigate competing value systems [7, 16, 17, 19, 57].

CB feedback highlighted how language shapes empowerment and inclusion throughout the curriculum. For example, renaming the “Advocacy” module to “Championship” illustrated how subtle linguistic shifts can recalibrate cultural tone—echoing research showing that non-stigmatizing terminology enhances psychological safety and engagement [58, 59]. This process of co-creation reflects broader principles of trauma-informed facilitation and community-driven implementation, where shared interpretation transforms descriptive feedback into actionable guidance [60]. Through the integration of quantitative prioritization and qualitative deliberation, the CB process we followed demonstrates how mixed methods can operationalize equity and produce practice-ready interventions [34, 61].

After reviewing the findings from the co-development process described above, the CB Members who are listed as co-authors on this manuscript (GG, AC, HO, SP, RRS, and VS) emphasized the importance of grounding Module 2’s content in an integrated framework that connects social determinants of health with recovery capital. Framing recovery capital through the structural conditions that shape health (e.g., housing stability, employment opportunities, and social connection) aligns with research highlighting the intertwined social, structural, and relational dimensions of recovery [62, 63]. This integration reinforces calls for multi-level interventions that link peer-delivered care with organizational and policy supports addressing systemic inequities. By embedding social determinants of health, recovery capital, and relational harm reduction, within eLearning and ECHO sessions, PATH will position training as empowerment rather than compliance—an approach consistent with evidence that peer-led education and mentorship enhance confidence, resilience, and fidelity to harm-reduction principles [64, 65].

The Cultural Exchange Survey provided an additional layer of confirmation that the CB functioned as a psychologically safe and collaborative environment [40, 66]. High mean ratings across all domains indicated strong alignment between CB Members and researchers. Open-ended feedback emphasized appreciation for equitable facilitation, time sensitivity, and opportunities for peer-only discussion. These findings echo prior studies showing that when PWLLE perceive genuine respect and shared power, collaboration quality and project retention improve substantially [38]. Breakout sessions and peer-led portions of meetings were particularly valued, highlighting that structural design elements of meetings are as important as the substantive content under discussion.

As in other participatory studies, ensuring consistent engagement throughout our CBPR process presented notable challenges [49]. Fluctuating participation, technology barriers, and competing demands reflected structural realities rather than a lack of commitment among CB members. Addressing these barriers required adaptive facilitation, equitable compensation, and flexible participation options—strategies recognized as essential for sustaining engagement in CBPR. Collectively, these adaptations underscore that responsiveness and flexibility are not merely logistical adjustments but active mechanisms of equity within participatory research [67]. Although our CB was relatively small, its membership represented diverse harm-reduction and recovery settings across the United States, and this representiveness enhances the contextual breadth and potential generalizability of PATH.

The next step in this work is to pilot PATH within two Recovery Community Organizations (RCOs). This pilot will assess the acceptability, appropriateness, and feasibility of PATH as a PRS workforce intervention, as well as satisfaction with its eLearning and ECHO training components. Findings from this pilot and continued CB partnership will inform refinements to PATH and may lead to additional modifications to enhance its usability and fit across diverse organizational contexts. The revised intervention will then guide the design of a subsequent stepped-wedge clinical trial evaluating PATH’s impact on workforce outcomes and service quality among PRS employed at ten additional RCOs.

## CONCLUSIONS

This early-phase study demonstrates how centering PWLLE through a CBPR process can produce a culturally grounded, practice-ready harm reduction training that reflects both scientific rigor and community wisdom. The co-development process is marked by shared decision-making, reflexive adaptation, and sustained representation across stages and illustrates how collaboration with PWLLE can strengthen intervention design and implementation readiness. Findings from this formative phase lay the groundwork for future testing of PATH’s feasibility and impact on workforce outcomes among PRS who deliver harm reduction services, organizational practices, and service quality across diverse recovery and harm-reduction settings.

## Figures and Tables

**Table 1:** Community Board Applicant and Selected Member characteristics

Demographics	Applicants (*N* = 89)	Invited for Follow-up Call (*n* = 23)	Selected Community Board Members (*n* = 8)
Age (Mean/SD)	44.97 (10.32)	47.35 (9.15)	43.13 (7.04)
Gender	N (%)		N (%)
*Male*	33 (37.1)	9 (39.1)	4 (50.0)
*Female*	51 (57.3)	12 (52.2)	4 (50.0)
*Other*	4 (4.5)	2 (8.7)	--
Race			
*White*	66 (74.2)	14 (60.9)	6 (75.0)
*Black or African American*	13 (14.6)	4 (17.4)	
*American Indian or Alaska Native*	2 (2.2)	2 (8.7)	1 (12.5)
*More than one race*	3 (3.4)	2 (8.7)	1 (12.5)
*Unknown*	5 (5.6)	1 (4.3)	--
Ethnicity			
*Latino*	13 (14.6)	5 (21.7)	1 (12.5)
*Not Latino*	71 (79.8)	17 (73.9)	7 (87.5)
*Unknown*	5 (5.6)	1 (4.3)	--
US region located			
*Northeast*	17 (19.1)	4 (17.4)	2 (25.0)
*Midwest*	22 (24.7)	6 (26.1)	1 (12.5)
*South*	36 (40.4)	6 (26.1)	2 (25.0)
*West*	14 (60.1)	7 (30.4)	3 (37.5)

Notes: Details regarding recovery status and harm reduction experience are omitted due to privacy concerns regarding the small Community Board size.

**Table 2.** Average prioritization scores for eLearning modules and lessons

Module topic	Mean (SD)^1^	Lesson topic	Mean (SD)^1^	Revised after group discussion
1. Embracing the harm reduction approach	3.88 (0.33)			No change
		1a. History of harm reduction	2.86 (0.83)	No change
		1b. Harm reduction principles	3.43 (0.73)	No change
		1c. Harm reduction and stages of change	3.14 (0.64)	Relational harm reduction^2^
		1d. Compatibility of harm reduction with the 12 steps	2.57 (0.90)	No change
2. Integrating harm reduction into participant recovery plans	3.63 (0.48)			No change
		2a. An overview of harm reduction strategies	3.57 (0.49)	No change
		2b. Motivational interviewing and harm reduction	3.29 (0.45)	Stages of change and harm reduction
		2c. Talking openly about drug use to identify high-risk behaviors	3.71 (0.45)	No change
		2d. Setting harm reduction goals	3.57 (0.49)	No change
		2e. Celebrating any positive success	3.29 (0.45)	No change
3. Supporting PRS to do harm reduction work	3.63 (0.48)			No change
		3a. Harm reduction-friendly policies	3.57 (0.49)	No change
		3b. Employee supervision	3.71 0.45)	No change
		3c. Identifying and addressing community barriers to harm reduction work	3.29 (0.70)	No change
		3d. Speaking with community stakeholders about harm reduction	3.43 (0.73)	No change
4. Advancing harm reduction^2^	3.25 (0.83)			No change
		4a. Introduction to being a harm reduction champion^2^	3.29 (0.88)	An introduction to being a harm reduction champion
		4b. Strategies for promoting harm reduction^2^	3.29 (0.45)	Harm reduction champion strategies and activities
		4c. Supporting Participants’ Voices^2^	3.71 (0.45)	Supporting participants to be champions of their personal recovery

1 Scale from 1/“not important” to 4/“extremely important”

2 Topic was not included in the originally funded grant application.

**Table 3:** ECHO prioritization scores and summary of open-ended feedback

ECHO training topic	Mean (SD)	Revised topics
1. Relational harm reduction	3.63 (0.48)	1. No changes
2. Managing boundaries with service participants	3.63 (0.70)	2. Managing boundaries with clients (will integrate self-care)
3. Selfcare	3.13 (0.78)	
4. Supporting colleagues to deliver harm reduction	2.88 (0.78)	3. Supporting colleagues to deliver harm reduction: Developing creative solutions together
5. Harm reduction with participants experiencing homelessness	3.63 (0.48)	4. No changes
6. Low-threshold treatment and Medication Assisted Recovery (MAR)	3.50 (0.71)	5. Low-threshold treatment and medications
7. Harm reduction in rural and low-resource areas	3.13 (0.60)	6. Harm reduction in low-resource areas
8. Harm reduction with criminal-legal system-involved participants	3.38 (0.70)	7. No changes
n/a	n/a	8. Harm reduction policy and legal structure

Notes: Scale from 1/“not important” to 4/“extremely important.”

**Table 4.** Cultural Exchange Survey Results

	Post Module Prioritization (*n* = 7)		Post ECHO Training Prioritization (*n* = 6)		Change
	*mean*	sd	*mean*	sd	*mean*
***Self-directed questions***					
I believe I have a clear understanding of what this collaboration is trying to accomplish.	5.9	0.4	5.8	0.4	0.0
I feel committed to this work.	5.7	0.5	5.8	0.4	0.1
I believe I am devoting the right amount of time and energy to maintain this collaboration.	5.4	0.8	5.3	0.5	−0.1
During the time I have been on the Board, I feel that my contributions have made an impact.	5.4	0.5	5.5	0.8	0.1
**Composite score**	**5.6**	**0.6**	**5.6**	**0.6**	**0.0**
***Fellow board member-directed questions***					
I believe we are working well together.	5.9	0.4	5.8	0.4	0.0
I believe we respect one another.	5.9	0.4	5.8	0.4	0.0
I believe my fellow Board members are committed to this work.	5.6	0.8	5.8	0.4	0.3
**Composite score**	**5.8**	**0.5**	**5.8**	**0.4**	**0.1**
***Research team-directed questions***					
I believe we are working well together.	5.9	0.4	5.8	0.4	0.0
I believe we respect one another.	5.7	0.5	5.8	0.4	0.1
I believe we trust one another.	5.7	0.5	5.8	0.4	0.1
I believe the research team is devoting the right amount of time and energy to maintain this collaboration.	5.9	0.4	5.7	0.5	−0.2
I believe the research team communicates openly with me.	5.9	0.4	5.8	0.4	0.0
I believe I communicate openly with the research team.	5.7	0.5	5.8	0.4	0.1
I believe the research team is committed to this work.	5.9	0.4	5.8	0.4	0.0
I believe the research team is learning something from me.	5.6	0.5	5.8	0.4	0.3
I believe I am learning something from the research team.	5.7	0.5	5.8	0.4	0.1
I believe the research team is changing their perspectives about something because of me.	5.4	0.8	5.8	0.4	0.4
I believe I am changing my perspective about something because of the research team.	5.1	0.9	5.7	0.5	0.5
I believe the research team is open to my concerns or wishes.	5.9	0.4	5.8	0.4	0.0
I believe that I am open to the concerns or wishes of the research team.	5.9	0.4	5.8	0.4	0.0
**Composite score**	**5.7**	**0.5**	**5.8**	**0.4**	**0.1**
***Additional Chair-Recommended Questions***					
I believe the community board selection process demonstrated respect for the perspectives of people with lived experience.	5.9	0.4	5.8	0.4	0.0
I would like the opportunity to engage in the continued research activities of this community board work (learning how to publish research, inform policy, etc.)	5.7	0.8	5.8	0.4	0.1

Notes: Questions asked using a scale of 1/“Not at all” to 6/“A great deal”

## Data Availability

All materials developed for the study are available upon request.

## Abbreviations

CB: community board
CBPR: community based participatory research
PATH: Peer Advanced Training in Harm reduction
PRS: peer recovery specialist
PWLLE: people with lived/living experience
SUD: substance use disorder
